# Fabrication of Beltlike Fibers by Electrospinning

**DOI:** 10.3390/polym10101087

**Published:** 2018-09-30

**Authors:** Yan-Qing Liu, Chun-Hui He, Xiao-Xia Li, Ji-Huan He

**Affiliations:** National Engineering Laboratory for Modern Silk, College of Textile and Engineering, Soochow University, 199 Ren-ai Road, Suzhou 215123, China; 20165215014@stu.suda.edu.cn (Y.-Q.L.); elysalxx@163.com (X.-X.L.)

**Keywords:** two-dimensional material, nanoparticle, dropping process, surfactants and dispersing agents, Taylor cone, mathematical model, graphene, molecule junction

## Abstract

Electrospinning is always used to fabricate one-dimensional nanofibers. Cylindrical fibers are formed during the spinning process due to the minimal-surface principle. However, when the moving jet has high rigidity, which can counteract the surface tension for a minimal surface, beltlike fibers can be obtained. Using the Hall–Petch effect, the rigidity of the moving jet can be greatly enhanced by adding nanoparticles. Polyethylene glycol with a nanometric crystallite size of 4 nm and ZrO_2_ nanoparticles are used as additives in the experiment, a theoretical analysis is carried out, and the theoretical predictions are verified experimentally.

## 1. Introduction

Electrospinning [1,2,3] has been widely used to fabricate one-dimensional fibers, though some special morphologies can be obtained, for example, micro-/nanoparticles, unsmooth fibers, porous fibers, and beaded fibers [4]. Morphology greatly affects the surface energy or products’ properties, and much attention has been paid to control fibers’ morphology [4,5,6,7,8,9,10,11,12]. Recently, beltlike fibers [13,14,15,16,17], bamboolike fibers [18], and crimped fibers [19,20] were reported, and their potential applications have been attracting much attention from various communities. Xu et al. [16] fabricated high-performance poly(p-phenylene) (PPP)-based polymer nanofiber belts that possessed high mechanical strength, remarkable thermal stability, excellent chemical resistance, and unique electrical and photoelectrical characteristics owing to the high rigidity of macromolecular backbones. Lu et al. [17] used monoclinic-phase VO_2_ nanoparticles with a diameter of 30–50 nm as additive in electrospinning, and they obtained ellipse-like or beltlike structural fibers. Liu et al. [18] obtained FeVO_4_ nanobelts with a width of about 400 nm by a simple electrospinning process, followed by a calcination process, and they found that crystallite size and the surface area of FeVO_4_ nanobelts were distinctly affected by the calcination temperature, and FeVO_4_ nanobelts had excellent photocatalytic properties. Chen et al. [19] and Huang et al. [20] used ash as an additive to fabricate beltlike and crimped fibers by bubble electrospinning. The morphology of obtained fibers greatly affects their thermodynamic compatibility [7,21,22,23,24] and their filtration properties, especially for the removal of heavy-metal ions [25,26,27].

In this paper, we unveil the mechanism for fabrication of beltlike fibers, and an experiment is carefully designed to verify our theoretical analysis.

## 2. Taylor Cone

**Figure 1:**
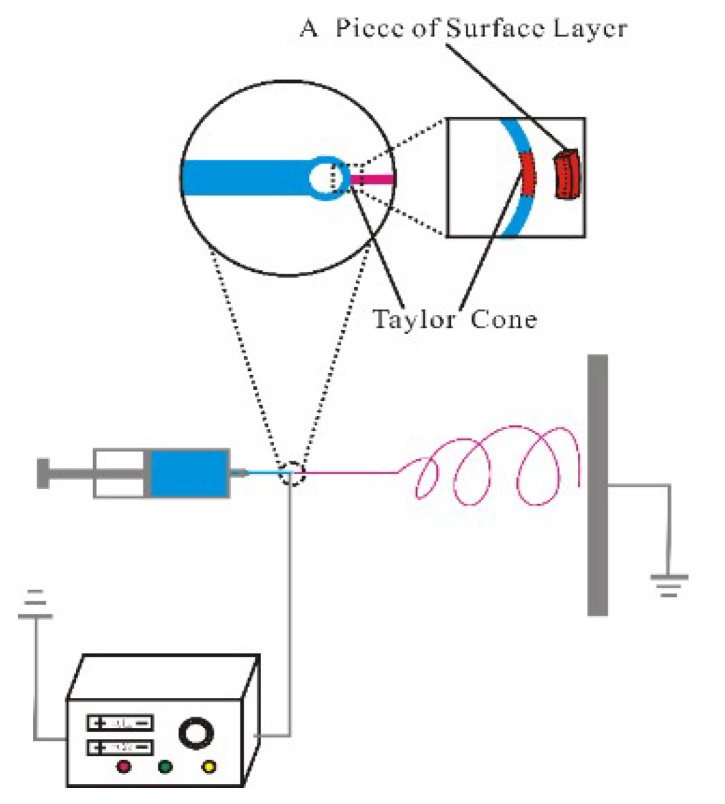
A control volume on the surface of Taylor cone.

Under a high electrostatic field, Taylor cone is a spherelike shape at the needle tip. During electrospinning, a Taylor cone is first formed. When the electrostatic force is high enough to overcome the Taylor cone’s surface tension [4], a jet is ejected. Consider a piece of surface layer of the Taylor cone before its ejection as illustrated in Figure 1. Considering a control volume, ignoring its inertia force due to the extremely slow extension of the Taylor cone and ignoring its circumferential motion, we can obtain the following normal force balance:

(1) EqA=T

where *E* is the electrostatic-field intensity, *q* is the surface charge density, *A* is the surface area, and *T* the viscous resistance.

As qualitative analysis, we assume that the motion of the control volume in Figure 1 is the Newtonian flow, and the viscous resistance acting on the control-volume section is:

(2) $$T=A\mu \frac{du}{dx}=A\mu \frac{u_{outer}-u_{inner}}{h}$$

where μ is the viscous coefficient, d*u*/d*x* is the normal velocity gradient, $u_{outer}$ and $u_{inner}$ are respectively the normal velocities of the outer surface and inner surface, and *h* is the thickness of the control volume.

Before a jet is formed, $u_{outer}$ is extremely small, while the value of $u_{inner}$ depends upon the flow field in the Taylor cone, which is a very complex process. Due to some perturbation, when $u_{outer}-u_{inner}$ is small enough, this piece of surface layer is ejected by the electrostatic force.

During the ejecting motion, the ejected piece forms a cylindrical fiber according to the minimal-surface theory [6] as we observe in almost all electrospinning process. If the moving jet in the electrospinning is rigid enough, the minimal-surface action is partly counteracted. A high concentration of a polymer solution is always highly viscoelastic, which enhances the rigidity of the moving jet.

## 3. Minimal Surface vs. Hall–Petch Effect

As discussed above, for most cases, electrospinning can fabricate one-dimensional smooth fibers due to request of the minimal surface or the minimal energy. The minimal-surface principle is the main factor in forming a sphere bubble in everyday observations, as well as in cylindrical fibers in electrospinning. However, under some constraints, noncylindrical fibers can be produced by electrospinning. The constraints include remarkably fast solvent evaporation, rigidity of the moving jets, and perturbation motion in Taylor cones. In this paper, in order to produce beltlike fibers, we focused on enhancing the rigidity of the moving jet in electrospinning to counteract the minimal surface by either increasing the concentration of the spun solution or adding nanoparticles.

Nanoparticles in a moving jet in the electrospinning process can greatly enhance rigidity by Hall–Petch strengthening [28,29,30], which has been widely used in material science. The Hall–Petch effect can be written in the form:

(3) $$\sigma =\sigma_{0}+\frac{k}{d^{\beta }}$$

where σ can be the elastic modulus or strength, $\sigma_{0}$ is its bulk’s property, *k* is a material constant, *d* is the mean grain size, and β is a scaling parameter. In most cases, β = 1/2 for qualitative analysis.

According to Equation (3), adding nanoparticles into the spun solution can greatly improve the rigidity of the moving jets that are ejected from the Taylor cone; this counteracts the minimal surface to form a beltlike fiber.

## 4. Experiment Design

A bisolvent system using dichloromethane (DCM) and dimethyl acetamide (DMAC) as solvents was prepared with different weight ratios. Polylactic acid (PLA, molecular weight:100,000) particles were bought from Shenzhen Esun Industrial Co. Ltd. Shenzhen, China and used without further purification. PLA particles were put into the bisolvent system in a sealed beaker, and the mixture was then magnetically stirred using a magnetic stirrer (HJ-6A, Gongyi Yuhua Instrument Co. Ltd. Gongyi, China) under ambient temperature until a uniform and transparent solution was obtained. Concentrations of PLA solution were listed in Table 1. In order to fabricate beltlike fibers, (Polyethylene Glycol)-400 (PEG 400) and ZrO_2_ nanoparticles (Shanghai ling feng chemical reagent co. LTD, Shanghai, China) were used as additives. Table 2 shows the spinning parameters in our experiment.

ZrO_2_ nanoparticles and PEG-400 with a diameter of about 4 nm are good additives in our experiment, which have a better Hall–Petch effect [30,31,32] on the fibers than other fillers with larger diameters.

The electrospinning setup was the same as that in our previous publications. The prepared solutions (Samples A, B, and C, listed in Table 1) were respectively put into a 10 mL syringe.

## 5. Results and Discussion

**Figure 2:**
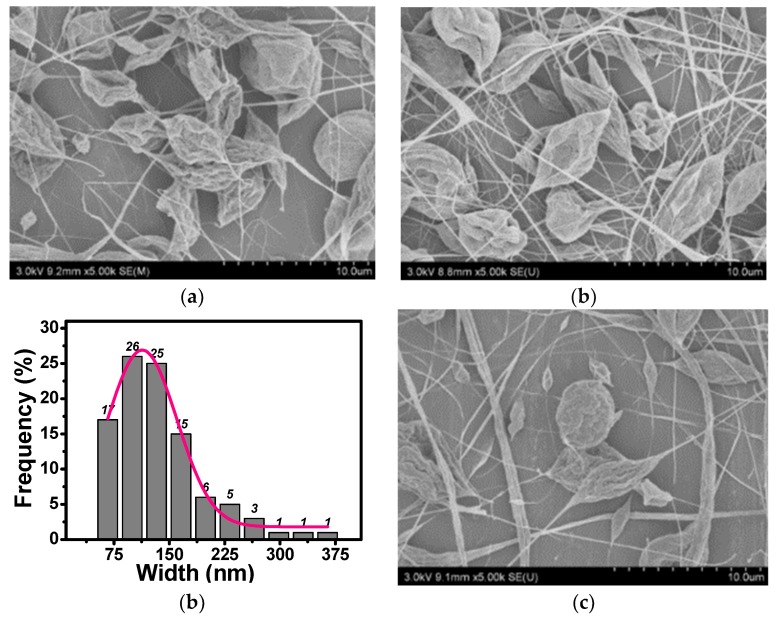
SEM illustrations for cases without additives. (**a**) Sample 1; (**b**) Sample 2; (**c**) Sample 3.

**Figure 3:**
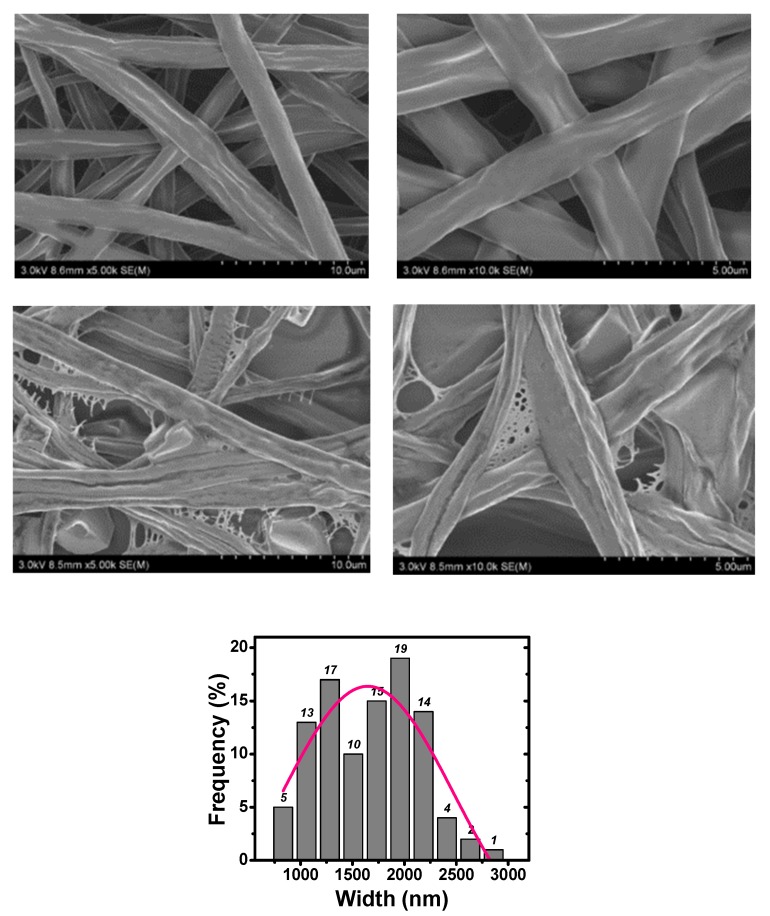
SEM illustration for Sample 4.

**Figure 4:**
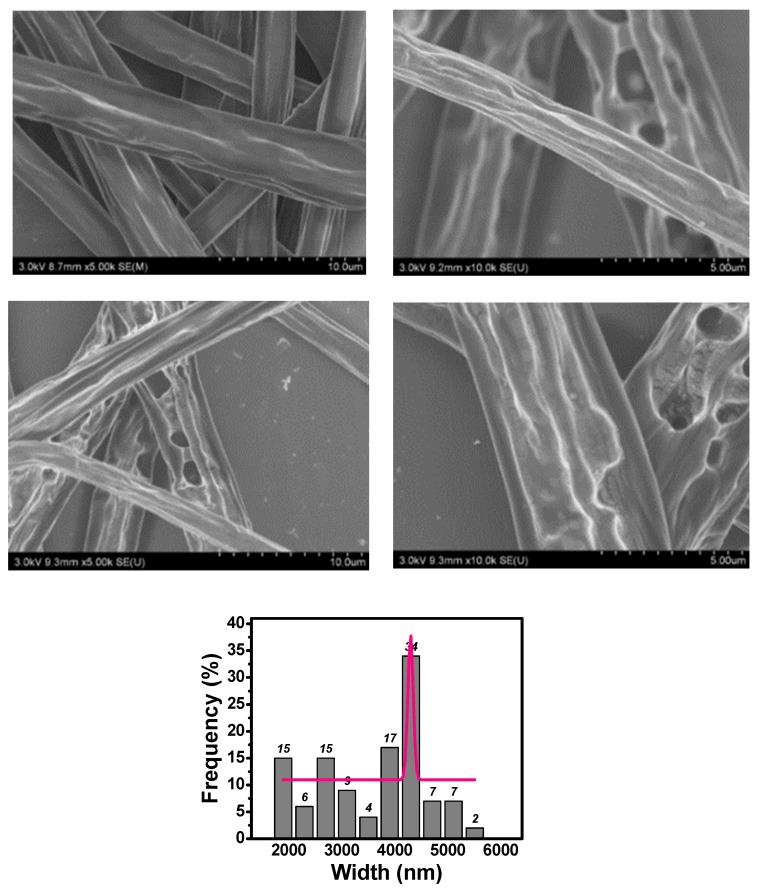
SEM illustration for Sample 5.

SEM illustrations for Samples 1–5 are given in Figure 2, Figure 3 and Figure 4, respectively. When neither zirconium dioxide nor polyethylene glycol were used as additives in the electrospinning, we could fabricate smooth fibers; fiber diameter depends upon many factors like applied voltage, polymer concentration, and receptor’s distance.

PLA is a semirigid polymer, and performs like petroleum-based plastics. This mechanical property is suitable for the fabrication of noncylindrical fibers by electrospinning.

It can be seen from Figure 2 that unsmooth beads were obtained. However, when PEG was added, beads disappeared completely, and beltlike fibers were obtained as shown in Figure 3. The formation of beads was also due to the minimal-surface principle, the Hall–Petch effect counteracted the surface tension, and beads or fibers could not be formed, as seen from Figure 3. When a ZrO_2_ nanoparticle was added to the PLA/PEG solution, the width of the beltlike fibers was larger than those without the ZrO_2_ nanoparticle.

**Figure 5:**
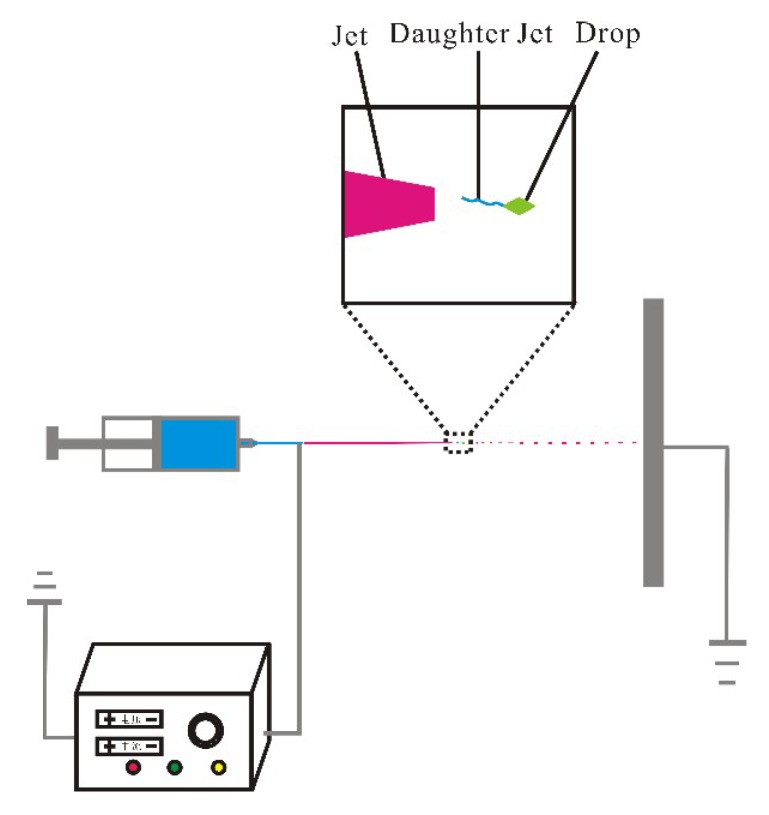
Dropping in a moving jet in electrospinning.

It is obvious that fibers could be obtained, but we can also observe irregular shapes and some beltlike fibers in Figure 3 and Figure 4. Due to the high viscosity of the PLA solution, the moving jet had high viscous resistance. This means that the moving jet decelerated fast at the initial stage and when acceleration became zero or very small, e.g., d*U*/d*x* = 0 or d*U*/d*x*, where *U* is the velocity of the moving jet. When *U* became extremely small, viscous resistance disappeared completely or became extremely small. Under such a condition, a drop is ejected from the jet due to inertial force, and daughter jets form during the dropping process. The drops finally form a sphere due to the minimal-surface principle. However, because of the high rigidity of the drops, a rhombuslike shape could be obtained, which linked with fibers formed from the daughter jets as illustrated in Figure 5.

If we assume that the moving jet is a Newtonian flow, the viscous resistance reads:

(4) $$T_{jet}=\pi r^{2}\mu \frac{dU}{dx}$$

where μ is viscous coefficient, *r* is the radius of the jet, and *U* is the velocity of the moving jet.

Due to the high viscosity of the solution, the jet decelerated remarkably, and when d*U*/d*x* ≤ 1, e.g., the moving jet’s velocity stayed almost unchanged, viscous resistance disappeared completely or became extremely small. Under such a condition, due to the inertial force, a drop is ejected from the jet, and daughter jets might form during the dropping process, as seen from Figure 5. For most cases, the drops form a sphere due to the minimal-surface principle. However, due to the high rigidity of the PLA fluid, which partly counteracts the minimal surface, a rhombuslike shape is formed.

According to Equation (2), the normal velocity of the Taylor cone is proportional to the flow-supply ratio or the applied voltage:

(5) $$u_{outer}\propto Q$$

(6) $$u_{outer}\propto V$$

According to Equation (2), viscous resistance is proportional to the normal velocity of the Taylor cone:

(7) $$T\propto u_{outer}$$

That means a higher flow-supply ratio or a higher applied voltage, resulting in higher viscous resistance for a piece of surface layer ejecting from the Taylor cone. As a result, a small fragment is formed that leads to either smaller fiber-/beltlike fiber or smaller spherelike shapes.

In order to enhance the rigidity of the moving jet, we can increase concentration of the PLA solution or add nanoparticles into the solution. It was reported that TiO_2_ nanoparticles in the PLA solution could produce porous fibers with good photocatalytic capability [33]. Magnesium oxide (MgO) nanoparticles could greatly reinforce the PLA fiber membrane that was used for food packing [34]. Another widely used additive is PEG [35,36], which is a polyether compound with many applications, from industrial manufacturing to medicine; its chemical formula can be written as C2nH4n + 2On + 1. PEG-400 can be used as a surfactant and dispersing agent. The nanometric crystallites of PEG-400 are about 4 nm. In this experiment, PEG was used as an additive. According to the Hall–Petch effect, small nanometric crystallites can greatly enhance the rigidity of the moving jet to counteract the minimal surface. As a result, beltlike fibers can be obtained as shown in Figure 3.

Concentration of the PLA solution greatly affects the morphology of the beltlike fibers. A higher concentration results in higher viscous resistance and higher rigidity. As a result, a higher voltage is needed and wider belts can be produced, as shown in Figure 4.

We use Sample 2 and Sample 4, given in Table 2, for comparison. The width of the beltlike fibers can be estimated using Equation (3). We assume that

(8) L∝σ

where *L* is the average width of the beltlike fibers. By Equation (3), we have

(9) $$\frac{L_{4}-L_{0}}{L_{2}-L_{0}}={\left(\frac{d_{2}}{d_{4}}\right)}^{\beta }$$

where Subscripts 2 and 4 imply Sample 2 and Sample 4, respectively. As qualitative analysis, we assume that the average grain size inversely scales with the average concentration of the PVA and PEG mixture. According to Reference [32], β=3/2. That means

(10) $$\frac{d_{2}}{d_{4}}=\frac{C_{4}}{C_{2}}=\frac{0.4+2}{0.4}=6$$

That means

(11) $$\frac{L_{4}-L_{0}}{L_{2}-L_{0}}={\left(\frac{2.4}{0.4}\right)}^{3/2}=14.6$$

This prediction is very close to the experimental data: 1657/137 = 12.1.

From Figure 2, we can see that some fibers were already broken for Samples 1–3 without any additives. The fibers might be broken under small perturbation during the spinning process, so fibers without PEG or nanoparticles as additives are extremely brittle. However, no broken fibers were observed for Sample 4; that implies that the addition of PEG leads to enhancement of the fiber’s strength. 

## 6. Conclusions

Nanofiber belts always have unusually high mechanical strength; hence, they could be used in special areas such as filtration media and separators in batteries and supercapacitors. A beltlike fiber can also be used as a two-dimensional material as a substitute of graphene in many advanced applications. For example, it can be served as an electrode in a molecule junction.

In this paper, for the first time ever, we give a theoretical analysis to produce beltlike fibers. The rigidity of the moving jet is the key factor affecting the morphology of the products. At low concentration, fibers, irregular shapes, and beltlike fibers can be formed. When the concentration increases, high voltage is needed and wide beltlike fibers can be obtained. Rigidity can be greatly enhanced by additives, and additives are an effective way to control the width of the beltlike fibers. The size of the additives also greatly affects the morphology of the beltlike fibers.

## Figures and Tables

**Table 1:** Polylactic acid (PLA) solutions with different concentrations.

Solution Sample	PLA (g)	Dichloromethane (DCM) (g)	Dimethyl Acetamide (DMAC) (g)	Polyethylene Glycol (PEG) (g)	ZrO_2_ (g)	Concentration (%)
A	0.4	17.64	1.96	0	0	2%
B	0.4	17.64	1.96	2	0	2%
C	1	15.57	1.73	2	0.2	5%

**Table 2:** Spinning parameters.

Samples	Voltage (kV)	Receptor’s Distance (cm)	Solution Supply Ratio (mL/h)	Solution Sample	Belt Width (nm)
1	15	17	0.1	A	-
2	15	17	0.2	A	137.59 ± 11.91
3	17	17	0.1	A	-
4	15	17	0.2	B	1657 ± 92.86
5	20	17	0.2	C	3493 ± 208.41
